# Natural vertical transmission of dengue virus in Latin America and the Caribbean: highlighting its detection limitations and potential significance

**DOI:** 10.1186/s13071-023-06043-1

**Published:** 2023-11-28

**Authors:** Mario A. J. Golding, Simmoy A. A. Noble, Nadia K. Khouri, Rhaheem N. A. Layne-Yarde, Inshan Ali, Simone L. Sandiford

**Affiliations:** 1 Department of Basic Medical Sciences, Pharmacology and Pharmacy Section, Faculty of Medical Sciences, The University of the West Indies, Mona, Kingston, Jamaica; 2 Department of Microbiology, Faculty of Medical Sciences, The University of the West Indies, Mona, Kingston, Jamaica; 3 College of Health and Wellness, Department of Health Sciences, Barry University, Miami Shores, FL 33161 USA; 4 Microbiology Laboratory, Memorial Healthcare System, Hollywood, FL 33021 USA; 5 Mosquito Control and Research Unit, The University of the West Indies, Mona, Kingston, Jamaica

**Keywords:** Vertical transmission, Transovarial transmission, Dengue virus, *Aedes aegypti*, *Aedes albopictus*, Caribbean, Latin America

## Abstract

Dengue continues to be a major public health concern in Latin America and the Caribbean with many countries in the region having experienced drastic increases in the incidence of dengue over the past few years. Dengue virus is predominantly transmitted by the bite of an infected female *Aedes aegypti* mosquito via a process called horizontal transmission. However, the virus may also be transmitted from an infected female mosquito to her offspring by vertical transmission, which occurs via viral invasion of the ovary either at the time of fertilization or during oviposition. In this way, mosquitoes may become dengue virus infected before ever encountering a human host. While some researchers have reported this phenomenon and suggested it may serve as a reservoir for the dengue virus in nature, others have questioned its epidemiological significance because of the low frequency at which it has been observed. Several researchers have either altogether failed to detect it or observed its occurrence at low frequencies. However, some studies have attributed these failures to small sample sizes as well as poor sensitivities of screening methods employed. Therefore, an overview of the occurrence, significance and limitations of detection of vertical transmission of dengue virus in *Aedes* mosquitoes in nature within Latin America and the Caribbean will be the focus of this review.

**Graphical Abstract:**

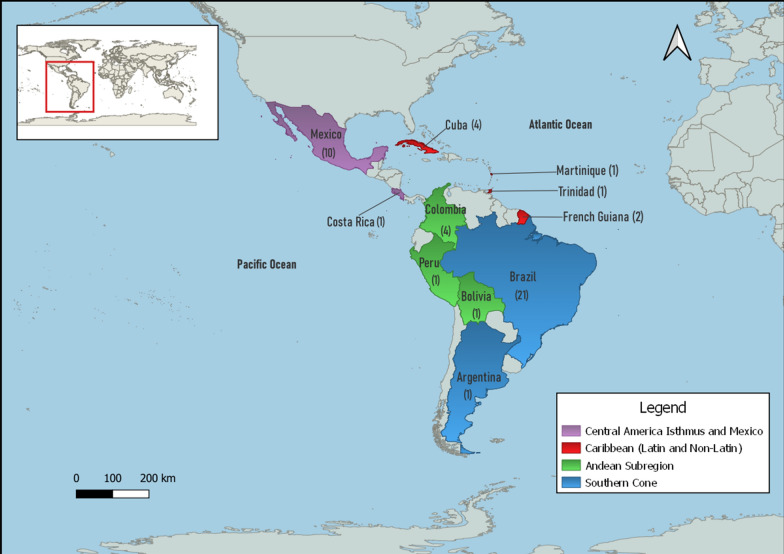

## Background

Dengue infection is caused by dengue virus (DENV), which is a single-stranded positive-sensed RNA virus belonging to the Flaviviridae family, genus Flavivirus [1]. The virus comprises four distinct serotypes, DENV-1, DENV-2, DENV-3 and DENV-4, which are transmitted to individuals mainly via the bite of an infected female *Aedes aegypti* or to a lesser extent female *Ae. albopictus* mosquitoes [2]. Infection by any of the four serotypes mainly results in classical dengue fever, which is usually mild and self-limiting. However, in some cases, the disease may progress to more severe and life-threatening forms such as dengue hemorrhagic fever (DHF) and dengue shock syndrome (DSS) [3, 4].

Dengue is now widely distributed in more than 100 countries in the tropical and subtropical regions of the world [5], where it places a significant socioeconomic burden on these areas [6]. In Latin America and the Caribbean (LAC), the economic impact of dengue is estimated to exceed US$ 3 billion annually, mainly due to loss of productivity, medical expenses and the cost of vector control programmes [7]. The ever-increasing geographic dispersal of dengue globally may be attributed to explosive population growth, urbanization, inadequate vector control and increased international travel and trade, potentially resulting in further spread of both vector and virus [8, 9].

Dengue infection continues to be the most significant arthropod-borne viral disease plaguing humankind with an estimated annual incidence of 100–400 million infections worldwide [2]. In 2019, the Americas recorded their largest number of reported cases of dengue in history with a total of 3,181,171 cases reported in the region, of which 44.5% were laboratory confirmed. This is in stark comparison to 757,082 reported cases (28.4% laboratory confirmed) in 2018 [10]. Despite being overshadowed by the COVID-19 pandemic, dengue remained relevant in the Americas in 2020 and 2021, where a total of 2,331,792 cases (43.3% laboratory confirmed) and 1,269,004 cases (41.5% laboratory confirmed) were reported respectively [10].

Dengue is predominantly transmitted in a human-mosquito-human cycle referred to as horizontal transmission (HT). When an individual is first infected with DENV, an immune response occurs which produces antibodies specific to that DENV serotype thus providing lifelong serotype-specific immunity. However, secondary infection by another DENV serotype results in enhanced infection via a phenomenon known as antibody-dependent enhancement [11, 12]. With multiple dengue serotypes circulating in LAC [10], it poses a considerable threat to individuals within this region since it increases the likelihood of developing DHF/DSS.

A female mosquito generally becomes infected with the virus when it acquires a blood meal from a viremic individual. The virus first infects the midgut of the mosquito and thereafter spreads to other tissues such as the salivary glands and reproductive tract during an extrinsic incubation period of 8–12 days, after which it can be transmitted horizontally to other individuals following subsequent feeding [13]. Dengue virus may also be transmitted vertically from an infected female mosquito to her offspring [14]. Vertical transmission (VT) may occur by either transovarial transmission, in which the virus infects germinal tissues of the female including oocytes or through trans-ovum transmission, which occurs during fertilization or by viral infection of the fully intact mature eggs during oviposition [15–17].

In the laboratory, VT of DENV may be confirmed by detecting the virus in the offspring of orally or intrathoracically inoculated females [17, 18], whereas in nature VT is assumed when the virus is detected in the immature mosquito stages or male mosquitoes [19]. It has been suggested that through VT, arboviruses such as DENV may be maintained in circulation during unfavorable conditions for vector activity such as in the absence of a vertebrate host or during the dry summer season [14, 16]. While VT of dengue has been shown to occur in nature, the frequency at which it occurs and its likely significance for the epidemiological status of dengue, particularly in the Caribbean, is not fully understood. Therefore, this article will seek to review the literature on VT within LAC with the aim of highlighting potential gaps in VT detection and its possible significance within the region.

### Data collection

Searches were conducted in PubMed, Lilacs and Google Scholar databases using a combination of the keywords: “vertical transmission,” “transovarial transmission,” “dengue,” “*Ae. aegypti*,” “*Ae. albopictus*,” “Caribbean” and “Latin America.” All English studies retrieved were read and evaluated. Studies in Spanish, French or Portuguese were converted to English using the Google translator tool. All studies investigating the natural occurrence of VT of DENV in various stages of field-collected *Ae. aegypti* and *Ae. albopictus* were included. Studies were excluded if they only examined dengue infection in field-caught adult females or investigated VT of DENV in mosquitoes under laboratory conditions. A total of 47 studies from 11 countries met the inclusion criteria for this review with 44 of them obtained after database searches and an additional three studies identified after a reference review.

Studies were grouped according to the assays used to detect VT of DENV. All relevant details such as information on collection period, number of pools screened, positive pools, total number of samples used and infection rate were included in this review once available.

### Evidence of vertical transmission in Latin America and the Caribbean

**Fig. 1:**
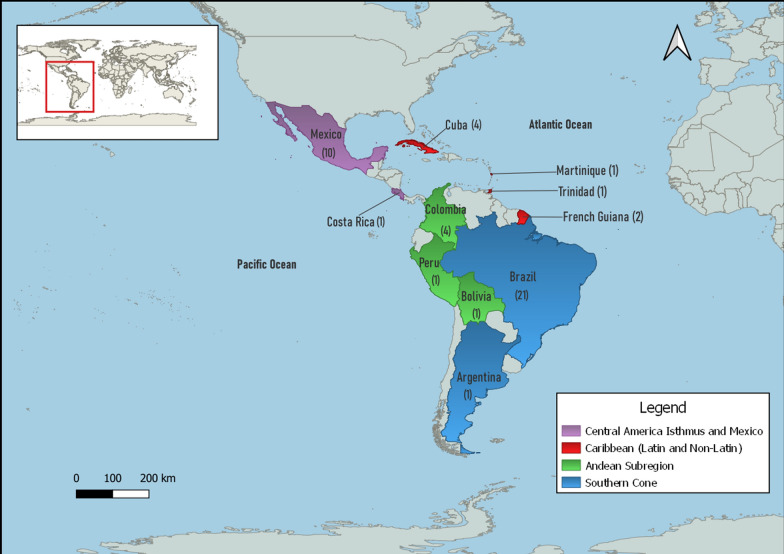
Natural vertical transmission of dengue virus in Latin America and the Caribbean. Distribution of studies by region reporting natural vertical transmission of dengue virus throughout Latin America and the Caribbean. Regions based on the Pan American Health Organization open data portal PLISA Health Information Platform for the Americas. Map created using QGIS 3.28.1

Vertical transmission of dengue virus has been extensively investigated within several countries in the LAC region (Fig. 1). The majority of studies occurred in Brazil and Mexico, where researchers have employed numerous techniques in their quest to demonstrate the occurrence or lack thereof of VT in nature.

### Detection of VT using immunofluorescent complement fixation or hemagglutination assays

Many of the earlier VT studies utilized immunofluorescent assay (IFA) in their evaluation of the phenomenon within the LAC region [20–26]. In most cases, to maximize the probability of virus detection using this assay, virus amplification was first performed using *Aedes pseudoscutellaris* (AP-61), *Ae. albopictus* (C6/36) or Vero cell lines. For example, in a study conducted in Trinidad [20] which was the first documented case of VT in LAC, researchers used IFA and complement fixation (CF) to screen 10,957 reared adult *Ae. aegypti* mosquitoes for DENV. These specimens were obtained from field immature stages collected from locations with confirmed dengue cases. Despite successfully identifying DENV-4 in 1 of 158 pools tested (Table 1), the observed minimum infection rate (MIR; per 1000) was 0.09, which was low (< 1 per 1000 individuals), although collections were made during a period of increased human infections of dengue.

**Table 1:** Natural vertical transmission of dengue in *Aedes* mosquitoes in Latin America and the Caribbean

Region	Species	Stage examined	No. of specimens	Positive pools/no. of tested pools	Screening method	Serotype (s) detected	Infection rate (per 1000)	Reference
Caribbean								
Cuba (Havana)	*Aedes aegypti*	Larvae & pupae	270	3/9	RT-PCR; sequencing	3	11.1 (MIR)	[31]
Cuba (Havana)	*Aedes aegypti*	Larvae & pupae	4, 102	37/111	RT-PCR; sequencing	1, 2, 3, 4	9.02 (MIR)	[32]
Cuba (Havana)	*Aedes aegypti*	Larvae	270	4/9	RT-PCR	1, 2, 3, 4	14.81 (MIR)	[70]
				9/9	RT-LAMP	N/A	33.33 (MIR)	
Cuba (Havana)	*Aedes albopictus*	Larvae	450	4/15	RT-PCR; nested PCR	3	8.88 (MIR)	[33]
French Guiana	*Aedes aegypti*	Eggs	2392	2/201	Isolation in AP-61 cells; indirect IFA	2, 4	0.84 (MIR)	[21]
		Larvae	2270	1/145		2	0.44 (MIR)	
French Guiana	*Aedes aegypti*	Eggs	3435	2/323	Isolation in AP-61 cells; indirect IFA	4	0.58(MIR)	[22]
		Larvae	4078	1/244			0.25 (MIR)	
Martinique	*Aedes aegypti*	Reared adults	N/A	1/101	RT-qPCR	N/A	N/A	[34]
Trinidad & Tobago	*Aedes aegypti*	Reared adults	10, 957	1/158	Isolation in AP-61 cells; IFA and CF	4	0.09 (MIR)	[20]
Central America Isthmus and Mexico								
Costa Rica	*Aedes albopictus*	Adult males	60	1/3	RT-PCR; sequencing	N/A	N/A	[35]
Mexico (Acapulco)	*Aedes aegypti*	Reared and field collected adult males	2, 200	2/93	4-plex real time RT-PCR	1	N/A	[37]
Mexico (Central and Southern States)	*Aedes aegypti*	Reared adult males	10, 620	6/354	Isolation in C6/36 cells; IFA; RT-PCR	1, 2, 3	2.52 (MIR)	[43]
		Reared adult females	10, 770	48/359				
Mexico (Guerrero)	*Aedes aegypti*	Field collected adult males	6, 594	6/424	RT-PCR	3, 4	N/A	[39]
Mexico (Nuevo Leon)	*Aedes albopictus*	Reared adults	1, 280	1/68	RT-PCR; nested PCR	N/A	N/A	[38]
Mexico (Oaxaca)	*Aedes aegypti*	Reared adult females	860	4/43	RT-PCR; snPCR	2, 3, 4	N/A	[36]
Mexico (Quintana Roo)	*Aedes aegypti*	Larvae	N/A	3/291	RT-PCR; snPCR	2	0.32 (MLE)	[40]
Mexico (Reynosa)	*Aedes albopictus*	Adult males	647	1/ N/A	Isoloation in C6/36 and Vero cells; HA; IFA; RT-PCR	2, 3	N/A	[23]
Mexico (Sinaloa)	*Aedes aegypti*	Larvae	308	2/14	RT-PCR; SnM-PCR; sequencing	2	6.49 (MIR)	[41]
Mexico (Sinaloa)	*Aedes aegypti*	Larvae	672	15/36	RT-PCR; semi-nested PCR; sequencing	4	22.32 (MIR)	[42]
Mexico (Yucatán)	*Aedes aegypti*	Field collected males	1278	12^a^	RT-qPCR	N/A	N/A	[44]
Andean Subregion								
Bolivia (Santa Cruz)	*Aedes aegypti*	Reared adult males	635	11/46	RT-PCR; snPCR	1, 3	17.32 (MIR)	[45]
		Reared adult females	748	3/51			4.01 (MIR)	
Colombia (Antioquia)	*Aedes aegypti*	Field collected males	1552	0^a^	IFA	N/A	N/A	[26]
Colombia (Antioquia)	*Aedes aegypti*	Reared adults	367	2/ N/A	RT-PCR	2	N/A	[47]
Colombia (Antioquia)	*Aedes aegypti*	Reared adults	1,497	131/400	RT-PCR; sequencing	1, 2, 3, 4	N/A	[48]
	*Aedes albopictus*	Reared adults	10	1/7			N/A	
Colombia (Cundinamarca)	*Aedes aegypti*	Larvae and pupae	N/A	N/A	RT-PCR; hnPCR	1, 2, 3, 4	N/A	[46]
Peru	*Aedes aegypti*	Reared adult females	N/A	N/A	RT-qPCR	2	N/A	[49]
Southern Cone								
Argentina (Misiones)	*Aedes aegypti*	Adult males	15	1/1	RT-PCR; nested PCR; sequencing	3	N/A	[50]
Brazil (Amazonas)	*Aedes aegypti*	Larvae	3, 956	70/146	RT-qPCR; sequencing	1, 2, 3, 4	17.70 (MIR)	[61]
Brazil (Amazonas)	*Aedes aegypti*	Field collected males	300	0/59^b^	RT-PCR	N/A	N/A	[67]
		Immature forms	1142					
Brazil (Bahia)	*Aedes aegypti*	Larvae	450	4/30	qPCR	N/A	N/A	[65]
			30	8^a^				
Brazil (Bahia)	*Aedes aegypti*	Larvae	20	8^a^	qPCR	N/A	N/A	[66]
Brazil (Ceará)	*Aedes aegypti*	Reared adult females	2, 005	1/41	Isolation in C6/C36 cells; IFA; RT-PCR; nested PCR; sequencing	2	0.50 (MIR)	[25]
	*Aedes albopictus*		212	2/6		2, 3	9.43 (MIR)	
Brazil (Mato Grosso)	*Aedes aegypti*	Reared adult males	351	5/26	SnM-PCR; sequencing	4	14.2 (MIR)	[58]
		Reared adult females	407	3/24			7.4 (MIR)	
Brazil (Mato Grosso)	*Aedes aegypti*	Reared adults	4, 490	8/57	Isolation in C6/36 cells; RT-PCR; sequencing	4	2.1 (MLE)	[59]
	*Aedes albopictus*		296	2/15			7.0 (MLE)	
Brazil (Mato Grosso)	*Aedes aegypti*	Field collected adult males	1139	1/84	Isolation in C6/36 cells; RT-PCR; sequencing	4	9.92 (MLE)	[60]
Brazil (Minas Gerais)	*Aedes albopictus*	Larvae	1, 128	2/ N/A	Isolation in C6/36 cells; IFA; PCR	1	N/A	[24]
Brazil (Minas Gerais)	*Aedes aegypti*	Larvae	2, 241	76/163	RT-PCR; snPCR	1, 2	33.9	[51]
	*Aedes albopictus*		1, 241	35/72			28.2	
Brazil (Minas Gerais)	*Aedes aegypti*	Field collected adult males	100	1/10	RT-PCR; sequencing	3	10.0 (MIR)	[53]
		Larvae	5, 573	1/101			0.18 (MIR)	
Brazil (Minas Gerais)	*Aedes aegypti*	Larvae	1, 400	163/435	RT-PCR	1, 2, 3	138.6 (MLE)	[52]
	*Aedes albopictus*		17	5/10		2, 3	N/A	
Brazil (Minas Gerais)	*Aedes aegypti*	Larvae	945	4/54	RT‐PCR	N/A	N/A	[54]
Brazil (Recife)	*Aedes aegypti*	Reared adults	2, 972	17/139	RT-PCR; snPCR	1, 2, 3	5.72 (MIR)	[55]
Brazil (Rio Grande do Norte)	*Aedes aegypti*	Larvae and pupae	1,1 86	4/46	Nested RT-PCR	4	3.37 (MIR)	[63]
Brazil (Rio Grande do Norte)	*Aedes aegypti*	Field collected adult males	78	3/17	Nested RT-PCR; sequencing	3	N/A	[64]
Brazil (Rio de Janeiro)	*Aedes aegypti*	Field collected adult males	369	1^a^	RT-PCR; snPCR	1	N/A	[62]
Brazil (Roraima)	*Aedes aegypti*	Larvae	1172	0/44	RT-PCR; hnPCR	N/A	N/A	[68]
Brazil (São Paulo)	*Aedes albopictus*	Larvae	542	3/26	hnRT-PCR; sequencing	3	N/A	[56]
Brazil (São Paulo)	*Aedes albopictus*	Reared adult males	1790	2/N/A	RT-PCR; snPCR; sequencing	3	N/A	[57]
Brazil (São Paulo)	*Aedes aegypti*	Larvae	910	0/91	Nested qPCR	N/A	N/A	[69]

Regions based on the Pan American Health Organization open data portal PLISA Health Information Platform for the Americas

*MIR* minimum infection rate, *MLE* maximum likelihood estimation, *N/A* not Available, *PCR* polymerase chain reaction, *RT-PCR* reverse transcription polymerase chain reaction, *HA* hemagglutination assay, *IFA* immunofluorescent assay, *CF* complement fixation, *qRT-PCR* real-time/quantitative reverse transcription polymerase chain reaction, *qPCR* quantitative polymerase chain reaction, *snPCR* semi-nested polymerase chain reaction, *SnM-PCR* semi-nested multiplex RT-PCR, *hnPCR* hemi-nested polymerase chain reaction, *hnRT-PCR* hemi-nested reverse transcription polymerase chain reaction, *RT-LAMP* reverse transcription loop-mediated isothermal amplification, *C6/36* *Aedes albopictus* cell line, *AP-61* *Aedes pseudoscutellaris*

^a^Samples individually analyzed

^b^Includes pools for males and immature forms

Since the identification of VT in Trinidad, several other investigators have opted to employ IFA as their screening technique for investigating VT of DENV. For example, Fouque and Carinci [21] illustrated that VT of DENV occurred under natural conditions in *Ae. aegypti* mosquitoes in French Guiana where 2 of 201 pools of *Ae. aegypti* eggs (2392 eggs) tested positive for DENV-2 and 4 and 1 of 145 pools of larvae (2270 larvae) tested positive for DENV-2 with MIRs of 0.84 and 0.44 respectively. Likewise, in another study conducted in French Guiana, DENV-4 was observed in the immature stages of *Ae. aegypti* mosquitoes collected from locations with suspected dengue cases during an endemic period in which DENV-1, 2 and 4 were isolated from human cases [22]. The investigators identified DENV-4 in 2 of 323 pools of eggs (3435 eggs screened) and 1 of 244 pools of larvae (4078 larvae screened) with MIRs of 0.58 and 0.25 respectively.

In Mexico, Ibáñez‐Bernal et al. [23] screened adult *Aedes* mosquitoes (2986 *Ae. albopictus* and 2339 *Ae. aegypti*) collected from the field in Reynosa, Tamaulipas, during a dengue outbreak for DENV. Specimens were collected from sites with high vector densities as well as confirmed human dengue cases, pooled and examined for virus using cytopathic effect (CPE) in C6/36 and Vero cell culture and by hemagglutination assay (HA). Positive samples were then examined by IFA, for which one pool of 10 *Ae. albopictus* males was positive for DENV 2 and 3. These results were confirmed by reverse transcription polymerase chain reaction (RT-PCR). However, the infection rate was not determined.

Similarly, using IFA Serufo et al. [24] reported DENV-1 in two pools of field-collected *Ae. albopictus* larvae (1128 larvae) during a period of active dengue transmission in humans in Campos Altos City, Minas Gerais. However, the number of pools tested and the infection rate were not stated. Additionally, in Fortaleza, Ceará, 1 of 41 pools of reared *Ae. aegypti* mosquitoes were found to be infected with DENV-2 and in 2 of 6 pools of reared female *Ae. albopictus* were positive for DENV-2 and 3 during an epidemic in which there was circulation of DENV-2 and -3 in humans [25]. Interestingly, the study locations were chosen based on mosquito infestation rate with no association with dengue cases. The results were confirmed by RT-PCR and nested PCR, and the MIRs were 0.50 and 9.43 for *Ae. aegypti* and *Ae. albopictus* respectively [25].

In contrast, Romero-Vivas et al. [26] demonstrated that VT of DENV does not always occur in nature. In the study conducted in Colombia, the researchers were able to detect DENV-1 and DENV-2 in 24 individually tested randomly collected field *Ae. aegypti* females (2065 females) during a period in which both serotypes were present in human cases. However, the researchers failed to identify DENV in 1552 individually analyzed *Ae. aegypti* randomly collected field males using IFA. The researchers suggested that the negative results may be due to the lower sensitivity of the IFA technique compared to newer techniques such as PCR [26]. It must be noted, however, that since detection of DENV was made in the female specimens, failure to do the same in the male specimens may not only involve the sensitivity of IFA technique as suggested by the authors.

Although IFA is relatively inexpensive, the assay is time-consuming, requires special facilities and is vulnerable to subjective interpretation, making it unsuitable for large-scale dengue surveillance [27, 28]. Furthermore, as techniques for identifying DENV in mosquitoes have advanced tremendously, the sensitivity of IFA is lower than that of newer techniques such as PCR.

Two of the aforementioned studies also utilized CF [20] and HA [23] to screen for the VT of DENV. However, these assays are no longer routinely used because of detection issues stemming from their limited sensitivities, lack of specificity and inability to identify the infecting virus serotype [28–30]. The former assay is also labor intensive, time consuming and challenging to perform and requires highly trained personnel [29]. Research that has employed these techniques in the evaluation of VT has typically also used other assays such as IFA and PCR to compensate for their limitations.

### Detection of VT using polymerase chain reaction and related techniques

#### Caribbean

Over time as research efforts to elucidate the significance of VT on the epidemiology of dengue infection intensified, newer and more sensitive techniques such as PCR have become the predominant tool for VT detection. Polymerase chain reaction techniques have revolutionized how VT of dengue in mosquitoes is detected with its increased sensitivity, specificity and versatility of applications. The availability of a wide range of PCR techniques, including reverse transcription PCR (RT-PCR), real-time or quantitative RT-PCR (RT-qPCR), nested and semi-nested PCR, and multiplex PCR, has enabled the utilization of the different variations in VT investigations. For example, in Cuba, researchers have been able to support the claim of VT of DENV occurring in nature on multiple occasions using RT-PCR. First, DENV-3 was detected in three of nine pools of *Ae. aegypti* larvae and pupae (270 specimens) with an MIR of 11.1 [31] suggesting that mosquitoes infected through natural VT could be contributing to dengue dynamics as DENV-3 was a frequently isolated serotype from human infected cases. Second, all four dengue serotypes were confirmed to be circulating in nature when 37 of 111 pools of *Ae. aegypti* larvae and pupae (4102 specimens) collected from areas with high infestation rates tested positive for DENV with an MIR of 9.02. The highest occurrence was DENV-1 (45.9%) followed by DENV-3 (43.2%), DENV-2 (32.4%) and DENV-4 (8.1%) [32]. Detection of DENV-1 in the mosquito population and not in human cases during the study period suggests that mosquitoes may be acting as a reservoir, keeping this serotype in circulation until a future outbreak. Additionally, researchers from Cuba reported DENV in *Ae. albopictus* for the first time in the Caribbean [33]. In their study, DENV-3 was detected in 4 of 15 pools of field-collected *Ae. albopictus* larvae (450 larvae) screened using RT-PCR and nested PCR with an MIR of 8.88.

In Martinique, for example, researchers used RT-qPCR to demonstrate VT of DENV in 1 of 101 pools of emerged *Ae. aegypti* mosquitoes collected from locations with confirmed or suspected dengue cases during a concomitant outbreak of dengue and chikungunya [34]. The virus was also detected in 4 of 167 pools of field-captured females but was not found in any of the male specimens tested. However, the serotype and infection rate were not reported.

#### Central America Isthmus and Mexico

Elsewhere in the LAC, researchers have observed VT of DENV in mosquito specimens using different PCR techniques. For example, in Costa Rica, with the use of RT-PCR, DENV was identified in 1 of 3 pools of male bodies of *Ae. albopictus* (60 males) and in 8 of 32 pools of females (640 females) collected from a dengue-endemic area [35]. The DENV RNA was not detected in the corresponding pools of heads, suggesting that dissemination had not yet occurred, and the virus was restricted to the gut [35]. Neither serotype nor infection rate was specified in the study.

In Mexico, multiple findings of VT have been documented. First, in Oaxaca, Mexico, using RT-PCR and semi-nested PCR, researchers were unable to identify DENV in 31 pools of field-collected larvae (620 larvae) but were able to detect DENV-2, -3 and -4 in 4 of 43 pools of adult female *Ae. aegypti* (860 mosquitoes) reared from larvae collected from the field in areas with documented dengue cases [36]. All four dengue serotypes were reported in human infections during the study period, with DENV-1 being the predominant serotype. The researchers concluded that the detection of DENV-2, 3 and 4 in the mosquito specimens suggest that these mosquitoes could be playing a role, albeit limited in the active transmission of the virus, as DENV-1 was not detected in the vectors [36]. In Acapulco, Mexico, using 4-plex Real time RT-PCR, DENV-1 was found in 2 of 93 pools of reared and field adult *Ae. aegypti* males (2200 males) collected from areas with reported dengue cases during an epidemic period in which human infections were mainly caused by DENV-1 and DENV-2. However, the infection rate was not stated [37]. Dengue virus has also been reported in 1 of 68 pools of reared adult *Ae. albopictus* mosquitoes (1280 specimens) screened by RT-PCR and nested PCR in Nuevo Leon, Mexico [38]. However, the serotype and infection rate were not determined. In the same study, the researchers failed to detect the virus in 35 pools of field-collected adult *Ae. albopictus* (556 specimens) or in any of the pools of emerged (685 specimens) and field-collected adult *Ae. aegypti* mosquitoes (148 specimens). The study sites of the specimens screened were chosen based on mosquito abundance, the presence of *Ae. albopictus* and reported cases of dengue [38]. The presence of DENV-3 and -4 was detected by RT-PCR in 6 of 424 pools of field *Ae. aegypti* adult males (6594 males) obtained from sites with confirmed dengue cases in Guerrero, Mexico, with all four DENV serotypes circulating in human cases. However, the infection rate of the positive samples was not stated [39]. In Quintana Roo, Mexico, DENV-2 was identified by RT-PCR and semi-nested PCR in 3 of 291 pools of *Ae. aegypti* larvae collected from the field in areas with reported probable dengue cases with a maximum likelihood estimation (MLE; per1000) of 0.32 during a period in which DENV-1 and -2 were circulating within this region [40]. Similarly, in Sinaloa, Mexico, RT-PCR and semi-nested PCR were used to identify DENV-2 in 2 of 14 pools of *Ae. aegypti* larvae (308 larvae) [41] and DENV-4 in 15 of 36 pools of *Ae. aegypti* larvae (672) [42] collected from the field with MIRs of 6.49 and 22.32, respectively. Likewise, following the laboratory rearing of randomly collected eggs from the field in the Central and Southern Mexican states of Morelos, Veracruz, Oaxaca and Chiapas during an epidemic period, using IFA, researchers were able to identify DENV-1, -2 and -3 in pools of *Ae. aegypti* mosquitoes with an MIR of 2.52 [43]. Moreover, DENV has also been reported in *Ae. aegypti* males collected from the field in Yucatán, Mexico, during a period of low human transmission. From a total of 1278 adult male mosquitoes individually screened by RT-qPCR, 12 individuals were positive. However, neither the serotype nor infection rate was determined [44].

#### Andean Subregion

The occurrence of VT of DENV has also been reported in Bolivia, Colombia and Peru. In Bolivia, using RT-PCR and semi-nested PCR, DENV-1 and -3 were detected in 11 of 46 pools of adult male *Ae. aegypti* (635 males) and in 3 of 51 pools of adult female *Ae. aegypti* (748 females) mosquitoes reared from preimaginal stages during a dengue outbreak with MIRs of 1.73% (17.32; per 1000) and 0.40% (4.01; per 1000) respectively [45]. The specimens were collected from locations with reported dengue cases as well as locations randomly chosen. The authors concluded that the significance of VT of dengue virus in the epidemiology of the disease is underestimated, stemming from the fact that the first identification of the DENV-1 serotype in humans occurred almost a year later after it was detected in the vector [45]. In Colombia, RT-PCR and hemi-nested PCR have been employed in the identification of all four dengue serotypes in pools of *Ae. aegypti* larvae and pupae during a dengue outbreak. However, the total specimens, pools positive, pools tested and infection were not specified [46]. Likewise, using RT-PCR, DENV-2 was recorded in 2 pools of reared *Ae. aegypti* adults (367 specimens) [47] and DENV-1, -2, -3 and -4 in 131 of 400 pools of reared *Ae. aegypti* (1497 specimens) as well as in 1 of 7 pools of reared *Ae. albopictus* (10 specimens) [48]. No infection rate was mentioned for either study. In a study in Peru, using RT-qPCR researchers observed DENV-2 in six pools of adult female *Ae. aegypti* mosquitoes reared from immature specimens collected from the field in dengue outbreak areas. No information was available on the number of pools tested, the total specimens tested and the infection rate [49].

#### Southern Cone

In an Argentinian study, one of one pool of male *Ae. aegypti* (15 specimens) tested positive for DENV-3 by RT-PCR and nested PCR [50] prompting the researchers to suggest that VT could be maintaining the virus in circulation during inter-epidemic periods since the specimens were collected during the winter-fall period when no dengue cases were recorded.

Evidence of VT of DENV in the LAC region is heavily dominated by studies from Brazil. Several researchers have demonstrated the occurrence of VT in various areas of the country such as in the state of Minas Gerais. For example, Cecílio et al. [51] detected DENV in pools of *Ae. aegypti* and *Ae. albopictus* larvae collected from locations with suspected or confirmed dengue cases during a period of active transmission. Using RT-PCR and semi-nested PCR, the researchers identified DENV-1 and -2 in 76 of 163 pools of *Ae. aegypti* larvae (2241 larvae) and in 35 of 72 pools of *Ae. albopictus* larvae (1241) with MIRs of 33.9 and 28.2 respectively [51]. Pessanha et al. [52] observed DENV in pools of *Ae. aegypti* and *Ae. albopictus* larvae from Belo Horizonte during a period of active dengue transmission in humans. The researchers used RT-PCR to screen 1400 *Ae. aegypti* larvae grouped into 435 pools, of which 163 pools were positive for DENV-1, -2 and -3 with an MLE of 138.6. DENV-2 and -3 were also identified in 5 of 10 pools of *Ae. albopictus* larvae (17 larvae) [52]. Similarly, the presence of DENV-3 was detected in 1 of 101 pools of *Ae. aegypti* larvae (5573 larvae) and 1 of 10 pools of field-collected adult males (100 males) with MIRs of 0.18 and 10.0 respectively [53]. The specimens were collected from areas with high numbers of dengue cases and high rates of mosquito infestation. In the same study, 3 of 15 pools of *Ae. aegypti* adult females were also found to be infected with the virus with an MIR of 21.9. The researchers suggested that the role of VT in the maintenance of dengue in nature in Minas Gerais could be significant since they were able to identify DENV-3 in mosquitoes in the same period in which DENV-3 was the main serotype detected in humans. Furthermore, investigators in Ouro Preto and Ouro Branco, Minas Gerais, detected DENV in 4 of 54 pools of *Ae. aegypti* larvae (945 larvae) using RT-PCR during a period of active transmission in humans. However, the serotype and infection rate were not specified [54].

Elsewhere in Brazil, DENV-1, -2 and -3 were detected in 17 of 139 pools of adult *Ae. aegypti* (2972 specimens) reared from eggs collected from areas with confirmed dengue cases in the city of Recife [55]. The viruses were also found in 9 of 83 pools of field-collected adults *Ae. aegypti* (301 specimens). These results suggested that VT could be playing a major role in the transmission dynamics of dengue as all three serotypes which were detected in the mosquitoes were also found circulating in humans during the study period. In Santos, São Paulo, DENV-3 was reported in 3 of 26 pools of *Ae. albopictus* larvae (542 larvae) collected from areas where dengue outbreaks were reported [56]. Similarly, using RT-PCR and semi-nested PCR, DENV-3 was identified in two pools of reared adult male *Ae. albopictus* (1790 specimens) from São Paulo during an epidemic period. However, there was no mention of the specific infection rate for the male specimens [57]. Researchers on multiple occasions identified DENV-4 in reared *Aedes* mosquitoes in the State of Mato Grosso [58–60]. In the study by Cruz et al. [58] using semi-nested multiplex RT-PCR, DENV-4 was detected in 5 of 26 pools of reared adult male (351 specimens) and 3 of 24 pools of female (407 specimens) *Ae. aegypti* mosquitoes with MIRs of 14.2 and 7.4 respectively during a dengue outbreak in which all four dengue serotypes were detected in human cases, with DENV-4 being most frequent. Likewise, using RT-PCR, DENV-4 was identified in 8 of 57 pools of reared adult *Ae. aegypti* (4490 specimens) and 2 of 15 pools of reared *Ae. albopictus* (296 specimens) with MLEs of 2.1 and 7.0 respectively [59] and in a 1 of 84 pools of field collected adult *Ae. aegypti* males (1139 specimens) with an MLE of 9.92 [60]. In the Amazonas state, all four dengue serotypes were detected by RT-qPCR in 70 of 146 pools of *Ae. aegypti* larvae (3956 specimens) collected from areas with elevated infestation rates during an epidemic period with an MIR of 17.70 [61]. In Rio de Janeiro, DENV-1 was reported in 1 of 369 randomly collected field *Ae. aegypti* males individually analyzed by RT-PCR and semi-nested PCR during an epidemic. In the same period, 24 of 2469 individually analyzed field-collected *Ae. aegypti* females were positive for DENV-1, -2, -3 and -4 [62]. Moreover, in Rio Grande do Norte State, DENV-4 was identified in 4 of 46 pools of *Ae. aegypti* larvae and pupae (1186 specimens) using nested PCR with an MIR of 3.37 during an active transmission period in humans [63]. At the same time, DENV-1, -2 and -4 were detected in 21 of 111 pools of field-collected adult female *Ae. aegypti* (1293 specimens) with an MIR of 16.2 and also in 6 of 19 pools of adult *Ae. albopictus* (67 specimens) [63]. In another study in Rio Grande do Norte State, using the nested PCR, investigators recorded DENV-3 in 3 of 17 pools of field-collected adult male *Ae. aegypti* mosquitoes (78 specimens) and in 4 of 19 field collected female *Ae. aegypti* mosquitoes. However, the infection rate was not mentioned [64]. In Bahia, researchers reported DENV in 4 of 30 pools of field collected *Ae. aegypti* larvae (450 specimens) analyzed by qPCR during an inter-epidemic period as well as in 8 of 30 individually analyzed larvae hatched from field-collected eggs [65]. However, the serotype and infection rate were not mentioned. Likewise, investigators in Bahia were able to detect DENV using qPCR in 8 of 20 individually analyzed *Ae. aegypti* larvae collected during the rainy season from areas with dengue cases [66]. However, the serotype and infection rate were not determined. Despite the many reports demonstrating evidence of VT in Brazil, some studies have failed to support the claim. For example, researchers in Manaus, Amazonas, detected DENV-3 in 14 of 82 pools of female *Ae. aegypti* (374 specimens) using RT-PCR but were unable to identify the virus in male and immature specimens. The researchers failed to detect DENV in 59 pools of adult males and immature *Ae. aegypti* (300 males and 1142 immature forms) although the specimens were collected from areas with suspected dengue cases during a period of active dengue transmission [67]. While this suggested that VT of DENV was not playing a role in the transmission dynamics of dengue in this region, the researchers instead attributed the negative results to the small number of specimens collected during the study. Likewise, investigators were unable to detect DENV in 44 pools of *Ae. aegypti* larvae (1172 larvae) using RT-PCR and hemi-nested PCR in Roraima, Brazil [68]. The study failed to find evidence supporting VT of DENV despite the specimens having been collected in the rainy season from areas with high dengue incidence in humans and high *Ae. aegypti* infestation rates. There was no mention of any adult field specimens being collected or analyzed. The negative results led the investigators to conclude that VT of DENV occurs at a very low frequency; therefore, it is not likely to be an important mechanism by which the virus is able to persist in the environment [68]. Furthermore, researchers failed to detect DENV using nested qPCR in 91 pools of field-collected *Ae. aegypti* larvae in Taubaté, São Paulo, Brazil. No adult specimens were collected or screened. The negative results were observed despite the study being conducted during a dengue epidemic [69]. The authors, therefore, concluded that vertical transmission was not playing any significant role in maintaining the virus in the area under investigation.

### Reverse transcription loop-mediated isothermal amplification

Reverse transcription loop-mediated isothermal amplification (RT-LAMP) is a relatively newer technique that has recently been used by Cuban researchers to investigate VT of dengue [70]. The RT-LAMP technique is used to amplify a target DNA sequence with high specificity and sensitivity without using specialized instruments [71]. Piedra et al. [70] demonstrated the superior sensitivity of this technique compared to PCR when they reported DENV in 9 of 9 pools of *Ae. aegypti* larvae (270 larvae) with an infection rate of 33.33 after they were only able to detect DENV in 4 of 9 of these pools (MIR: 14.81) using RT-PCR. The ease of use, quick reaction time, efficiency and easy detection procedures of RT-LAMP assay make it a favorable new technique. However, a fundamental limitation is the necessity for accurate primer design [72]. Additionally, the greater the number of primers per target in LAMP, the greater the probability of primer-primer interactions [72]. While the variability in VT occurrence and infection rates being influenced by the chosen detection method was aptly demonstrated by Piedra et al. [70], more studies are necessary to determine if RT-LAMP is indeed a superior technique to PCR for the detection of VT of DENV.

### Estimation of vertical transmission in mosquitoes

As evident by the studies reviewed, the most frequently used method for estimating infection rate in pooled mosquito samples was the minimum infection rate (MIR; per 1000). The MIR is defined as the ratio of the number of positive pools to the total number of mosquitoes in the sample and relies on the assumption that in a positive pool, only one infected individual exists. Another method that was used to estimate infection rate in pooled mosquito samples was the maximum likelihood estimation (MLE; per 1000). The MLE is defined as the value of the proportion of infected mosquitoes, *P*, that maximizes the likelihood of n pools of size m to be virus positive, where *P* is the parameter for a binomial distribution [73]. The MLE is seen as a more accurate and robust measure of infection rate than MIR as it measures the infection rate itself and does not rely on the assumption that only one individual in the sample is infected. However, MLE has not been widely appreciated or applied by researchers in the estimation of infection rates [74].

The MIR is generally considered a useful measure of infection rate in situations where only a small portion of tested pools are positive, such as where the calculated MIR is < 1 per 1000 individuals or where the sample size is small [74, 75]. Under these conditions, there are no significant differences between estimates of MIR and MLE [75]. However, at high infection rates and large pool sizes, using MIR may lead to underestimation of the infection rate. While the MLE is more accurate than the MIR, it is vulnerable to fluctuations in the reactivity of pools of varying sizes, whereas the MIR remains constant regardless of which pool the infected individual is in [74]. As illustrated in the study by Gu et al. [74], the estimated infection rates in the VT studies reviewed in this article could also differ depending on the estimation method used. Using MIR could lead to underestimation of the true infection rate which would undervalue the significance of VT on the persistence of DENV in nature and its possible involvement in the dengue dynamics. Thus, using MLE may be more appropriate for estimating infection rates.

### Relevance of VT in nature

The disparity in reported results on VT throughout LAC continues to question its significance in dengue transmission in the region. As expected, the literature is dominated by reports of VT detected in specimens when analyzed by PCR. Using this method, investigators typically saw a greater infection rate in the tested specimens compared to IFA studies which potentially highlights the superior sensitivity of the PCR technique. For example, in the Cuban study by Gutiérrez‐Bugallo et al. [32], an MIR of 9.02 was recorded compared to an MIR of 0.25 in the IFA study by Fouque et al. [22], although a similar number of larvae were screened (4102 and 4078, respectively). On the other hand, in some instances, the higher infection rate observed with PCR was possibly due to the smaller number of tested specimens. Vilela et al. [53] reported an MIR of 10.0 but only 100 specimens were tested, which is lower than the number of specimens screened in any of the IFA studies. While PCR was the technique of choice for VT evaluations in the LAC as it allows for rapid detection of the dengue virus and is highly specific and sensitive, it is expensive and requires expertise skills and specialized equipment [29, 76]. Thus, it may not be suitable for VT evaluations in resource-limited countries.

Intriguingly, despite the use of this sensitive technique, multiple researchers have failed to detect VT during their experiments with many attributing this fact to insufficient numbers being screened. While this may have indeed contributed to the results obtained, one can argue that other factors must be considered when interpreting results. In some instances, sample collection protocols were not adequately described. While many state that collections were done during periods of high DENV transmission to humans and high mosquito infestations, the time lapse between reported cases and mosquito collections was not reported. Additionally, whether these collections were random or targeted was also not indicated.

Laboratory experiments conducted in well-controlled environments have shown that the filial infection rate of F1 progeny can vary widely. Rosen et al. [77] for instance reported that F1 progeny infection rate was affected not only by the species and geographic origin of mosquitoes but also the serotype and strain of the virus. Notably, some strains of *Ae. albopictus* transmitted DENV to their progeny more readily than strains of *Ae. aegypti* that were investigated [77]. It should therefore come as no surprise that significant variation regarding VT is seen throughout the region. It has also been shown that detection of infected progeny is influenced by the time interval between the initial infection and the day of oviposition [78]. When collecting immature stages from the field, regardless of whether this occurs randomly or in active transmission areas, it is impossible to know the time interval between the infection of the female and oviposition. As such, the outcome of VT studies may be inadvertently influenced by oviposition timing. Lastly, variables that influence the vector competence of mosquitoes such as insect-specific viruses (ISVs) may also influence the detection of VT of dengue [79]. It has been suggested that infection of mosquitoes with ISVs may result in superinfection exclusion, which is a phenomenon where the replication of the same or a similar virus is not supported in cells already infected with a virus [80]. This, like many other factors within the region, remains largely unexplored and requires further investigation.

## Conclusion

The collective data from research conducted in the LAC have confirmed the existence of VT of DENV in nature. At present, however, its epidemiological significance in disease transmission remains highly controversial. This is primarily because of the many knowledge gaps that persist in the region regarding mosquito populations and virus strains in addition to the lack of standardization with reporting. Therefore, the epidemiological significance of VT should not be discounted solely based on low filial infection rates reported in nature. Instead, there is a need for standardized methods to be developed and implemented to ensure that assays used for the detection of VT are consistent regarding the collection of samples, number of samples screened and testing methodology employed, thus allowing for better comparison across studies. Given the limited documentation on this phenomenon in the Caribbean, it is difficult to draw any conclusions, underscoring the need for greater research in this area. Overall, the discovery of VT of DENV in LAC emphasizes the importance of investigating its natural occurrence and suggests that its detection may serve as an early warning sign for future outbreaks, as shown in Bolivia [45].

Furthermore, higher MIR as observed in many of the studies from Cuba may have been a reflection of this country’s robust surveillance mechanisms, which emphasizes the need for improved surveillance systems and vector control strategies geared towards controlling the immature stages of *Aedes* mosquitoes as these stages could act as reservoirs for DENV, helping to maintain it in circulation.

## Data Availability

Not applicable.

## Abbreviations

VT: Vertical transmission
DENV: Dengue virus
DHF: Dengue hemorrhagic fever
DSS: Dengue shock syndrome
LAC: Latin America and the Caribbean
CF: Complement fixation
IFA: Immunofluorescent assay
PCR: Polymerase chain reaction
RT-PCR: Reverse transcription polymerase chain reaction
qPCR: Quantitative polymerase chain reaction
qRT-PCR: Real-time/quantitative reverse transcription polymerase chain reaction
RT-LAMP: Reverse transcription loop-mediated isothermal amplification
MIR: Minimum infection rate
MLE: Maximum likelihood estimation
